# Oral Antacid Use Is Negatively Associated with Serum Prealbumin Levels in Japanese Individuals Undergoing Health Checkups

**DOI:** 10.3390/nu16213715

**Published:** 2024-10-30

**Authors:** Chihiro Ushiroda, Kanako Deguchi, Risako Yamamoto-Wada, Hiroko Tanaka, Chisato Ono, Mitsuyoshi Yoshida, Masayoshi Sarai, Ryoji Miyahara, Hitomi Sasaki, Katsumi Iizuka

**Affiliations:** 1 Department of Clinical Nutrition, Fujita Health University, Toyoake 470-1192, Japan; chihiro.ushiroda@fujita-hu.ac.jp (C.U.); kanasakuran@gmail.com (K.D.); risako.wada@fujita-hu.ac.jp (R.Y.-W.); 2 Department of Dentistry and Oral-Maxillofacial Surgery, Fujita Health University, Toyoake 470-1192, Japan; htanaka@fujita-hu.ac.jp (H.T.); mitsuyoshi.yoshida@fujita-hu.ac.jp (M.Y.); 3 Department of Medical Technology, Fujita Health University Haneda Clinic, Tokyo 144-0041, Japan; chisato.ono@fujita-hu.ac.jp; 4 International Medical Center, Fujita Health University Hospital, Toyoake 470-1192, Japan; msarai@fujita-hu.ac.jp (M.S.); ryoji.miyahara@fujita-hu.ac.jp (R.M.); sasakih@fujita-hu.ac.jp (H.S.); 5 Food and Nutrition Service Department, Fujita Health University Hospital, Toyoake 470-1192, Japan

**Keywords:** albumin, ALB, prealbumin, PAB, vitamin B_12_, antacids, occlusal force, masticatory performance, *Helicobacter pylori*

## Abstract

Background/Objectives: The aim of this study is to investigate the association between physical and chemical digestion and nutrition markers (serum albumin (ALB), prealbumin (PAB), and vitamin B_12_ (B_12_) levels). Methods: During a detailed checkup at Fujita Health University, we examined the associations of physical (occlusal force, masticatory performance, and swallowing ability (via the 10-item Eating Assessment Tool, EAT-10)) and chemical (*Helicobacter pylori* (HP) eradication history, HP antibody levels, and oral antacid (proton pump inhibitors) use) digestion parameters with serum ALB, PAB, and B_12_ levels in 92 individuals (M:67, F:25). Results: Forty-eight percent of the participants were older than 65 years of age, 19% had decreased occlusal force, 3.2% had decreased masticatory strength, 3.2% had decreased swallowing function, 24% had a history of HP eradication, 23% were HP antibody positive, and 16% were taking oral antacid medication. Additionally, 14% and 11% of the patients had low serum ALB and PAB levels, respectively, and 14% of the patients had B_12_ deficiency. Multivariate analysis adjusted for age, sex, body mass index, and C-reactive protein levels revealed that there were no significant associations between the physical digestion parameters and the serum PAB, ALB, or B_12_ levels. On the other hand, there was a significant association between oral antacid use and PAB levels (β = −3.3, *p* = 0.04). Independent of physical or chemical digestion parameters, serum PAB and B_12_ levels were significantly associated with protein and B_12_ intake, respectively. Conclusions: Oral antacid use may decrease serum PAB levels, indicating protein synthesis.

## 1. Introduction

In Japan, the average life span has increased, and 13.2% of men and 22% of women older than 65 years are malnourished [1]. Malnutrition causes prolonged duration of hospitalization, decreased mobility, and increased infection risk, making it a serious problem for clinicians [2]. Malnutrition worsens patient prognosis, highlighting the importance of intervention before hospitalization [2].

Malnutrition arises from reduced dietary intake, malabsorption, increased nutrient losses or altered metabolic demands [3,4,5,6,7]. Oral function is also implicated in the development of malnutrition. For example, malocclusion, a condition in which the opposing teeth do not mesh normally, is a risk factor for malnutrition among community-dwelling frail elderly individuals [4,5,6]. In Korea, subjects with poor chewing ability have lower food intake [6]. Severe undernutrition occurs due to malabsorption and improves following refeeding [7]. Thus, malnutrition is caused by a decrease in oral function, digestion, and absorption.

Malnutrition is often accompanied by decreased protein synthesis, as indicated by decreased serum albumin (ALB) and prealbumin (PAB) levels [3,8]. Serum PAB and ALB levels are regarded as nutritional markers reflecting short- and long-term malnutrition, respectively [3]. With respect to short-term protein synthesis, rapid-turnover proteins such as PAB and retinol-binding proteins are often used because of their short half-life in the blood. Protein intake affects serum ALB and PAB levels. Moreover, ALB has long been used as a representative marker of malnutrition; however, serum ALB levels are easily affected by inflammation, and therefore, serum ALB and C-reactive protein (CRP) levels are known to be negatively associated with each other [3,9]. As protein levels are also affected by physical, chemical, and bacterial digestion, we considered whether disorders of physical and chemical digestion also have short- and long-term effects on plasma PAB and ALB levels.

Malnutrition is often also accompanied by vitamin B_12_ deficiency [10,11,12]. Vitamin B_12_ is used as a cofactor for enzymes that are involved in the synthesis of deoxyribonucleic acid (DNA), fatty acids, and myelin [10,11,12]. Vitamin B_12_ deficiency can cause peripheral neuropathy, clinical microcytic anemia, depression, atherosclerosis, and cognitive problems [10,11,12]. Vitamin B_12_ deficiency is a serious disorder that, if not treated, can lead to severe progressive neurological symptoms. The mechanism of vitamin B_12_ absorption involves chemical digestion, binding to intrinsic factors in the stomach, and cecal absorption; moreover, vitamin B_12_ is stored mainly in the liver [10,11,12]. The causes of vitamin B_12_ deficiency are very broad and include autoimmune disease, gastrectomy, the use of drugs such as antacids and metformin, insect infection, and increased consumption of thyroid hormones [10,11,12]. Malnutrition is also associated with vitamin B_12_ status [8,13]. Therefore, physical and chemical digestion decreases serum vitamin B_12_ levels as well as serum protein levels.

Although the relationships between nutritional status and albumin, prealbumin, and vitamin B_12_ concentrations have been extensively studied as described above, few studies have simultaneously measured physical and chemical digestion and evaluated their relationships with serum ALB, PAB, and vitamin B_12_ concentrations. In this study, we aimed to clarify whether physical or chemical digestion affects serum PAB, ALB, and vitamin B_12_ levels. Identifying the factors influencing these levels will promote the identification of appropriate subjects for supplying easily absorbable amino acids and vitamins.

## 2. Materials and Methods

### 2.1. Study Design and Participants

The study sample consisted of 201 individuals who had undergone a medical checkup at Fujita Health University International Medical Center from April 2022 to December 2022.

Among the 201 patients, 128 underwent a complete interview, were assessed for occlusion and chewing and swallowing function, and provided blood samples. Thirty-six patients (19 men and 17 women) taking vitamin supplements were excluded, and 92 patients (67 men and 25 women) not taking vitamin supplements were included in the study. The aim of this retrospective, cross-sectional observational study was to clarify the associations between serum nutrition markers (PAB, ALB, and vitamin B_12_ levels) and factors influencing physical and chemical digestion with respect to sex, age, body mass index (BMI), and CRP levels.

### 2.2. Food Frequency Questionnaire on Food Groups

During the medical examination, a food frequency questionnaire (FFQg) was used to collect dietary information [14]. The FFQg is one of the most widely used food intake frequency questionnaires in Japan [15].

### 2.3. Physical Examination

Data on age (years), sex, BMI (kg/m^2^), waist circumference (cm), handgrip strength (kg), sleep duration (h), and blood parameters (glycated hemoglobin A1c (HbA1c, %), estimated glomerular filtration rate (eGFR, mL/min/cm^2^), uric acid (UA, mg/dL), triglycerides (TG, mg/dL), high-density lipoprotein cholesterol (HDL-C, mg/dL), total cholesterol (TC, mg/dL), and non-HDL-C (mg/dL) were obtained at the Fujita Health University International Medical Center health checkup and linked to the FFQg data. Height, weight, waist circumference (cm), and handgrip strength (kg) were measured in the presence of a nurse. BMI was automatically calculated from height and weight. Sleep duration was self-reported, and the subjects simply stated the average sleep duration per week (e.g., six hours). Plasma creatinine, uric acid, and lipid (TC, TG, and HDL-C) concentrations were measured via a Hitachi LABOSPECT008 (Hitachi High-Tech Corporation, Tokyo, Japan), and HbA1c was measured via an A1c HA-8190 (Arkray, Kyoto, Japan). The eGFR was automatically calculated via the plasma creatinine level, age, and sex. Non-HDL-C concentrations were calculated from the TC and HDL-C concentrations. Blood samples were collected from non-fasting participants at approximately 16:00–17:00. The data are presented as the means (SDs). Titers of antibodies against *H. pylori* (HP) were measured via *H. pylori*-LATEX “SEIKEN”(Denka Ltx, Gosen, Niigara, Japan).

### 2.4. Chewing and Swallowing Functions

#### 2.4.1. Occlusal Force

**Figure 1:**
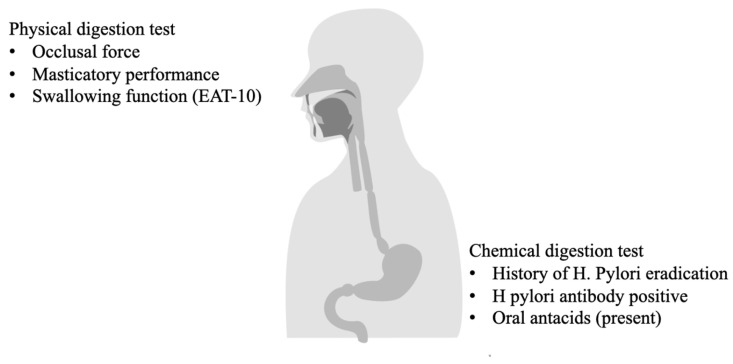
Summary of Physical and Chemical Digestion Test.

Using a pressure-sensitive film (Dental Prescale II, GC Corp., Tokyo, Japan) and a Biteforce Analyzer (GC Corp.), the occlusal force of the entire dentition was measured during 3 s of clenching in the occlusal–occipital fit position, and an occlusal force less than 500 N was defined as a reduced occlusal force [16]. The denture wearers underwent this measurement with their dentures in place (Figure 1).

Physical digestion test includes occlusal force masticatory performance, and swallowing function (EAT-10). The Chemical Digestion test includes History of *H. pylori* eradication, *H. pylori* antibody positive, and oral antacids use.

#### 2.4.2. Chewing Function

We used a chewability test (measuring the amount of glucose eluted during the chewing of a gummy jelly containing glucose) to evaluate the results. After 2 g of gummy jelly (Glucolam, GC Corp.) was freely chewed for 20 s, 10 mL of water was added, and the gummy and water were discharged into a filtration mesh. The amount of eluted glucose in the solution passing through the mesh was measured via a chewing ability test system (GlucoSensor GS-II, GC Corp.) to determine the eluted glucose concentration. The glucose concentration was measured via the GlucoSensor GS-II [17]. A glucose concentration of less than 100 mg/dL was considered indicative of masticatory dysfunction.

#### 2.4.3. Swallowing Function (EAT-10)

We assessed swallowing ability via the 10-item Eating Assessment Tool (EAT-10), and a total score of 3 or higher was considered to indicate decreased swallowing ability.

#### 2.4.4. History of HP Eradication, Positive Titers of HP Antibodies, and Use of Oral Antacids and Metformin

A history of HP eradication was confirmed via interviews. Patients with an antibody titer of 10 or higher were defined as HP antibody positive. Patients were considered to include both those currently infected with HP and those who had been infected in the past but were not currently infected. The use of oral antacids or metformin was confirmed via interviews. Oral antacid use was defined as the use of a proton pump inhibitor (PPI). Antacids are PPIs (out of 15, 4 esomeprazole, 2 bonoprazan, 1 rabeprazole, and 8 lansoprazole).

### 2.5. Statistical Analysis

To determine the effects of occlusal force, masticatory performance, the EAT-10 score, a history of HP eradication, HP antibody positivity, and oral antacid use on serum PAB, ALB, and vitamin B_12_ levels, multivariate linear regression models adjusted for age, sex, BMI, and CRP levels were constructed. Model 1 was adjusted for occupational force, nutrient (protein or vitamin B_12_) intake, sex, age, BMI, and CRP; Model 2 was adjusted for chewing function, nutrient (protein or vitamin B_12_) intake, sex, age, BMI, and CRP; and Model 3 was adjusted for swallowing, nutrient (protein or vitamin B_12_) intake, sex, age, BMI, and CRP.

To determine the effects of history of HP eradication, HP antibody positivity, and oral antacid use on serum PAB, ALB, and vitamin B_12_ levels, multivariate linear regression models adjusted for age, sex, BMI, and CRP levels were constructed. Model 1 was adjusted for history of *H. pylori* eradication, nutrient (protein or vitamin B_12_) intake, sex, age, BMI, and CRP; model 2 was adjusted for *H. pylori* Ab+ (antibody titer above 10 U/L), nutrient (protein or vitamin B_12_) intake, sex, age, BMI, and CRP; and model 3 was adjusted for oral antacids, each nutrient (protein or vitamin B_12_) intake, sex, age, BMI, and CRP. The data are presented as the means ± standard deviations (SDs). *p* < 0.05 was considered to indicate statistical significance. This study is an exploratory study, and the sample size was not calculated. Statistical analyses were performed via SPSS version 28.0.0.0 software for Mac (IBM Corp., Armonk, NY, USA).

## 3. Results

### 3.1. Background of the Study Sample

A total of 92 individuals (67 men and 25 women) were evaluated in this study, and their characteristics were as follows. The average age was 62.2 ± 14.3 years (Table 1). The percentage of individuals older than 65 years of age was 48% (*n* = 44). The average BMI of the participants was 23.9 ± 3.2, with 4% being underweight and 25% being overweight. The percentage of individuals with a low occlusal force (<500 N) was 19.4%. The percentage of individuals with masticatory performance (Glc ≦ 100 mg/dL) was 3.2%. The percentage of individuals with decreased swallowing capacity was 3.2%. The percentage of individuals with a history of HP eradiation was 24% (*n* = 22). The percentage of individuals with increased titers of HP antibodies was 23% (*n* = 21). The percentage of individuals with a history of taking oral antacids (PPIs) was 16% (*n* = 15). The total cholesterol level was 205.5 (34.1) mg/dL, the HDL-C level was 56.8 (17.5) mg/dL, the triglyceride level was 127.4 (81.5) mg/dL, and the HbA1c level was 5.94 (0.74) %. The percentage of individuals with a higher CRP level (≧1.0 mg/dL) was 4% (*n* = 4). Thyroid function (TSH, FT_4_, and FT_3_) was normal in all but one case. Thus, this sample included a wide range of age groups and had a slightly greater proportion of male participants. A very small number of participants with high CRP levels that potentially affected the serum ALB and PAB levels were also included.

### 3.2. Food Intake and Nutritional Deficiency of the Subjects

The participants completed the FFQg, and we checked their blood PAB and vitamin levels, which reflect decreased protein synthesis and vitamin deficiency (Table 2). The energy intake (1776.4 ± 346.8 kcal), fat intake (61.8 ± 15.8 g), carbohydrate intake (202.4 ± 51.3 g), and protein intake (66.9 ± 15.3 g) results were assumed to be underestimated because of the nature of the FFQg. The percentage of individuals with low serum ALB levels was 14% (*n* = 13), the percentage of individuals with low serum PAB levels was 11% (*n* = 10), and the percentage of individuals with low vitamin B_12_ intake was 5.4% (*n* = 5). Thus, the study sample included individuals with low serum ALB, PAB, and vitamin B_12_ levels.

### 3.3. Multivariate Linear Analysis: Physical Digestion

Physical digestion plays an important role in the absorption of several nutrients, especially amino acids and vitamin B_12_. We performed multivariate linear regression analysis to clarify the associations between physical digestion parameters (occlusal force, chewing function, and swallowing function) and several nutritional markers (PAB, ALB, and vitamin B_12_ levels).

First, we clarified the associations of serum PAB levels with occlusal force, chewing ability, and swallowing ability. In Model 1, PAB levels were not associated with occlusal force (*p* = 0.88) but were significantly associated with protein intake (β = 0.10, *p* = 0.01), sex (β = −4.01, *p* = 0.003), and CRP levels (β = −1.75, *p* = 0.02) (Table 3). In Model 2, PAB was not associated with masticatory performance (*p* = 0.15), whereas PAB was significantly associated with protein intake (β = 0.1, *p* = 0.007), sex (β = −3.9, *p* = 0.003), and CRP levels (β = −1.68, *p* = 0.03). In Model 3, PAB was not associated with the EAT-10 score (β = 1.7, *p* = 0.72) but was significantly associated with protein intake (β = 0.1, *p* = 0.008), sex (β = −4.0, *p* = 0.003), or CRP level (β = −1.77, *p* = 0.02).

In Model 1, the serum ALB concentration was not associated with occlusal force (*p* = 0.56) but was significantly associated with only the CRP concentration (β = −0.11, *p* = 0.01). In Model 2, the serum ALB concentration was significantly associated with masticatory performance (β = −0.001, *p* = 0.02) and the CRP concentration (β = −0.12, *p* = 0.006). In Model 3, PAB was not associated with the EAT-10 score (β = 0.02, *p* = 0.45) but was significantly associated with the CRP level (β = −0.11, *p* = 0.01).

In Model 1, the serum vitamin B_12_ level was not associated with occlusal force (*p* = 0.5) but was significantly associated with vitamin B_12_ intake (β = 12.3, *p* = 0.007), sex (β = 86.2, *p* = 0.006), and age (β = 3.2, *p* < 0.001). In Model 2, the serum vitamin B_12_ level was not associated with masticatory performance (*p* = 0.65) but was significantly associated with vitamin B_12_ intake (β = 12.7, *p* = 0.005), sex (β = 87.4, *p* = 0.005), and age (β = 3.3, *p* < 0.001). In Model 3, vitamin B_12_ was associated with the EAT-10 score (β = −1.5, *p* = 0.006), sex (β = 87.5, *p* = 0.005), and age (β = 3.3, *p* < 0.001).

Thus, occlusal force and the EAT-10 score were not associated with PAB, ALB, or vitamin B_12_ levels, although masticatory performance was very weakly associated with serum ALB levels.

### 3.4. Multivariate Linear Analysis: Chemical Digestion

Chemical digestion plays an important role in the absorption of several nutrients, especially amino acids and vitamin B_12_. Since HP infection and antacids are known to inhibit gastric acid secretion, we performed multivariate linear regression analysis to clarify the associations between several chemical digestion parameters and nutritional markers (PAB, ALB, and vitamin B_12_ levels).

In Model 1, PAB was not associated with HP eradication (*p* = 0.12) but was significantly associated with protein intake (β = 0.1, *p* = 0.006), sex (β = −4.0, *p* = 0.003), and CRP levels (β = −1.6, *p* = 0.034) (Table 4). In Model 2, PAB was not associated with HP antibody positivity (*p* = 0.65), age (*p* = 0.28), or BMI (*p* = 0.82), whereas PAB was significantly associated with protein intake (β = 0.1, *p* = 0.01), sex (β = −4.0, *p* = 0.003), and CRP levels (β = −1.8, *p* = 0.02). In contrast, in Model 3, PAB was associated with oral antacid use (β = −3.3, *p* = 0.04), protein intake (β = 0.1, *p* = 0.007), and sex (β = −4.1, *p* = 0.002). Thus, oral antacid use was significantly and negatively associated with PAB levels.

In Model 1, the serum ALB level was not associated with HP eradication (*p* = 0.93) but was significantly associated with only the CRP level (β = −0.11, *p* = 0.01). In Model 2, the serum ALB level was not associated with HP antibody positivity (*p* = 0.55) but was significantly associated with the CRP level (β = −0.11, *p* = 0.01). In Model 3, the ALB level was not associated with the use of oral antacids (*p* = 0.13) but was significantly associated with the CRP level (β = −0.10, *p* = 0.03). Thus, oral antacid use was significantly and negatively associated with PAΒ levels. In Model 1, vitamin B_12_ levels were not associated with HP eradication (*p* = 0.11), whereas vitamin B_12_ levels were significantly associated with vitamin B_12_ intake (β = 12.7, *p* = 0.005), sex (β = 87.8, *p* = 0.004), and age (β = 3.4, *p* < 0.001). Similarly, in Model 3, vitamin B_12_ levels were not associated with oral antacid use (*p* = 0.56), whereas vitamin B_12_ levels were significantly associated with vitamin B_12_ intake (β = 12.8, *p* = 0.005), sex (β = 88.0, *p* = 0.005), or age (β = 3.1, *p* = 0.003). Thus, serum PAB levels, but not serum ALB or vitamin B_12_ levels, were significantly associated with oral antacid use.

## 4. Discussion

In this study, we aimed to clarify whether physical or chemical digestion affects several nutritional markers (PAB, ALB, and vitamin B_12_ levels). Only oral antacid use was associated with decreased serum PAB levels. Importantly, protein synthesis may be suppressed in individuals taking antacids, even if they are ingesting protein.

In the present study, none of the variables related to physical digestion (occlusal force, masticatory performance, EAT-10) were significantly associated with plasma PAB, ALB, or vitamin B_12_ levels. Some studies reported that a decrease in occlusal force was significantly associated with decreased intake of vegetables; vitamins A, C, and B6; folate; and dietary fiber [18]. Since 83 individuals in our study had more than 20 teeth and normal occlusal force, plasma PAB or vitamin B_12_ levels may have been less affected by these physical digestion factors. It is possible that different results would have been obtained if these associations had been examined in a population with fewer than 20 teeth or with reduced occlusal strength. Thus, poor chewing and swallowing may not only lead to reduced protein intake [5] but may also affect overall digestion. In the present study, it was possible to analyze cases where the effects of physical digestion, such as swallowing and chewing, could be relatively excluded.

In the present study, serum PAB levels were significantly associated with oral antacid use (PPIs) and protein intake. In contrast, the serum ALB level was not associated with either oral antacid use or protein intake. Protein intake and antacids were important factors, which can be rephrased as amino acid absorption from the small intestine. PAB is a rapid-turnover protein and is used as a marker for protein synthesis, but the blood half-life of ALB is much longer than that of PAB [3,8]. Our results showed that plasma PAB levels were associated with oral antacid and protein intake after adjustment for sex, age, BMI, protein intake, and CRP. In contrast, the serum ALB level was not associated with either antacid or protein intake. Only the CRP level was associated with the serum ALB level. These findings suggest that, compared with the serum ALB level, the serum PAB level is a more sensitive marker of the effects of protein intake and antacids on protein absorption.

Unlike oral antacid use (PPIs), HP infection was not associated with PAB, ALB, or vitamin B_12_ levels. Chronic infection with HP may be associated with either decreased or increased acid secretion, depending on the severity and type of gastritis [19]. Considering that the inhibitory effect of antacids on gastric acid secretion was stronger than the effect of HP infection was, in medical interviews, it is important to confirm whether patients are using antacid medication.

In contrast, oral antacid use was not significantly associated with serum vitamin B_12_ levels. However, inhibiting gastric acid secretion, i.e., gastrectomy or antacid use, is known to inhibit vitamin B_12_ absorption [10,11,19,20]. The duration and dose of antacid also affects the absorption of vitamin B_12_ [21]. Two or more years of PPI or H2RA use were both associated with an increased risk for vitamin B_12_ deficiency [19]. Consuming more than 1.5 PPI pills/d was more strongly associated with vitamin B_12_ deficiency than was taking fewer than 0.75 pills/d [20]. Since vitamin B_12_ accumulates in the liver, the duration, amount, and type of antacid administration may be particularly important. Unfortunately, we did not explore the duration and dose of antacid administration. Therefore, we concluded that there was no association between antacid use and blood vitamin B_12_ levels in this study.

The serum vitamin B_12_ concentration was significantly positively associated with age and sex (female). PAB levels were only associated with sex (female), and ALB was not associated with age or sex. Another research group reported that women consume more whole grains, vegetables, and salty foods than men do, whereas men consume more meat than women do [21]. Our previous studies revealed that women consume less meat than fish [22]. Moreover, we reported that in both sexes, the fish intake frequencies of 40–50-year-olds and 50–60-year-olds were significantly greater than those of 20–30-year-olds [22]. In young underweight Japanese women, vitamin deficiency is common [23]. Considering that vitamin B_12_ is abundant in fish and in the liver [10], differences in food preferences (especially those of fish) by sex may affect PAB and vitamin B_12_ concentrations.

In the present study, we were the first research team to identify the associations between PAB levels and antacid use. Some papers reported that patients experienced few minor side effects of short-term PPI use, such as headache, rash, dizziness, and gastrointestinal symptoms, including nausea, abdominal pain, flatulence, constipation, and diarrhea. Infections, impaired absorption of nutrients (magnesium and vitamin B_12_), dementia, kidney disease, and hypergastrinemia-related side effects are also emerging as possible consequences of long-term use. One of the expected side effects of PPIs should be a decrease in protein synthesis reflecting serum PAB levels.

The finding that antacids and protein intake affect serum PAΒ may be beneficial for patients with more frequent PAB measurements, i.e., older patients who are malnourished and undernourished patients receiving enteral or intravenous nutrition. Particularly in cases where protein intake is low, such as in the elderly, there is concern that antacids may further reduce protein absorption, leading to reduced protein synthesis, as typified by PAΒ levels, and ultimately to pathologies such as sarcopenia. The association between protein intake and frequency of sarcopenia in patients on long-term oral antacids should be investigated in the future. Moreover, it is also possible that oligomeric formulae or elemental diets (ED) may have higher absorption rates than polymeric formulae in patients receiving tube feedings but also receiving antacids. Differences in protein synthesis capacity between different types of enteral nutrition in patients receiving antacids may be worth investigating in the future.

The study naturally has to consider the existence of confounding factors. Factors that may influence serum ALB and PAB include the degree of inflammation, thyroid function, liver function and renal function. CRP, ALB, and PAB levels were negatively associated with CRP levels. Under inflammatory conditions, interleukin (IL)-6 plays an important role in increasing the synthesis of acute-phase proteins, such as CRP, instead of suppressing ALB and PAB synthesis in the liver [9,24]. Thus, we included CRP as an independent variable in our multivariate analysis. If ALB or PAB is used as a nutritional marker, a normal CRP level must first be confirmed.

In addition, hyperthyroidism, chronic kidney disease, and liver dysfunction may affect serum prealbumin and albumin levels. For example, hyperthyroidism causes a decrease in both serum PAB and vitamin B_12_ levels. In this study, we did not include thyroid function as a cofactor; however, thyroid function was almost normal except in one individual. Similarly, renal and liver function was almost normal, and we did not include these factors as cofactors. Thus, thyroid, liver and renal function can be potential confounding factors, and we did not include thyroid, renal, or liver tests as independent variables in the multivariate analysis.

The limitations of this study are as follows. This was a retrospective observational study, and the cause and results remain unclear. However, given that gastric acid is required for protein digestion and that prealbumin reflects short-term nutritional status, it is reasonable to assume that the prealbumin concentration is positively associated with protein intake and negatively associated with oral antacid use. In contrast, to identify markers that reflect long-term nutritional impairment (e.g., ALB and vitamin B_12_ levels), information such as the duration of antacid administration is needed. Moreover, the group in our study included individuals who were willing to pay expensive fees for physical check-ups and were interested in their own health conditions. Since most of the individuals in this study had more than 20 teeth and no abnormalities in chewing or swallowing function, an evaluation of the associations of chewing and swallowing function with various nutritional markers should be performed in patients with chewing and swallowing dysfunctions. On the other hand, by studying patients with relatively preserved swallowing and chewing functions, as in the present study, the effects of chemical digestion (antacids) could be studied more clearly. Future studies in patients with impaired swallowing and masticatory function may need to exclude the effects of chemical digestion.

## 5. Conclusions

In conclusion, oral antacid use may decrease protein synthesis as the result of decreased protein digestion in the stomach. Particularly in elderly individuals and women, in addition to insufficient protein intake, decreased protein breakdown due to antacid use may inhibit protein absorption and suppress markers of protein synthesis, such as PAB. The incorporation of foods that improve protein digestion, such as tofu, yogurt, milk, white fish, ground chicken, and semi-cooked eggs, may be beneficial. Additionally, if these foods are not very effective, the use of supplements broken down into polypeptides or amino acids to compensate for indigestion caused by gastric acid may be desirable. Inquiring about the presence or absence of antacids in malnourished patients is important for successful nutritional therapy.

## Figures and Tables

**Table 1:** Background of the subjects without vitamins supplement.

	Total (*n* = 92)
Sex (%Male)	72% (*n* = 67)
Age (yo)	62.2 (14.3)
Age (≧65 yo)	48% (*n* = 44)
BMI	23.9 (3.2)
Under weight (<18.5)	4% (*n* = 4)
Normal weight	61% (*n* = 56)
Obese weight (≧25)	35% (*n* = 32)
Occlusal force (N)	993.6 (533.7)
Lower Occlusal force (<500 N)	19.4% (*n* = 18)
Masticatory performance (mg/dL)	200.9 (64.5)
Lower masticatory performance (<100 mg/dL)	3.2% (*n* = 3)
Swallowing function (EAT-10)	0.17 (1.17)
EAT-10 ≧ 3	3.2% (*n* = 3)
History of *H. pylori* eradication	24% (*n* = 22)
H pylori antibody positive (≧10 U/mL)	23% (*n* = 21)
Oral antacids (present)	16% (*n* = 15)
Metformin (present)	7.6% (*n* = 7)
Total cholesterol (mg/dL)	205.5 (34.1)
HDL cholesterol (mg/dL)	56.8 (17.5)
Triglyceride (mg/dL)	127.4 (81.5)
HbA1c (%)	5.94 (0.74)
eGFR (mL/min/1.73 m^2^)	71.3 (18.1)
CRP (mg/dL)	0.2 (0.72)
CRP ≧ 1.0 mg/dL	4% (*n* = 4)
TSH (μIU/mL)	1.89 ± 1.36
FT_4_ (ng/dL)	1.35 ± 0.27
FT_3_ (pg/mL)	3.18 ± 0.43

Data represented as Mean (SD).

**Table 2:** Food intake and nutrition deficiency of the subjects without vitamins supplement.

	Total (*n* = 92)
Energy intake (kcal)	1776.4 (346.8)
Fat intake (g)	61.8 (15.8)
Carbohydrate intake (g)	202.4 (51.3)
Protein intake (g)	66.9 (15.3)
Albumin (g/dL)	4.28 (0.29)
Lower albumin (<4 g/dL)	14% (*n* = 13)
Serum prealbumin (mg/dL)	28.9 (5.7)
Lower prealbumin (<22 mg/dL)	11% (*n* = 10)
Vitamin B_12_ intake (μg)	6.3 (3.0)
Lower vitamin B_12_ intake (<2.4 μg)	5.4% (*n* = 5)
serum vitamin B_12_ (pg/mL)	333.2 (139.1)
Vitamin B_12_ deficiency	14% (*n* = 13)

**Table 3:** Multivariate analysis of physiological digestion and serum prealbumin, albumin, and vitamin B_12_ levels.

	Dependent Variable:		Prealbumin			
	Model 1		Model 2		Model 3	
	β (95%CI)	*p*	β (95%CI)	*p*	β (95%CI)	*p*
Occlusal force (N)	0 (−0.002, 0.002)	0.88				
Masticatory performance (mg/dL)			0.01 (−0.01, 0.03)	0.15		
EAT-10					0.17 (−0.76, 1.11)	0.72
Protein intake (g)	0.10 (0.02, 0.17)	0.01	0.10 (0.03, 0.17)	0.007	0.10 (0.03, 0.17)	0.008
Sex (female)	−4.01 (−6.61, −1.41)	0.003	−3.91 (−6.48, −1.34)	0.003	−4.00 (−6.59, −1.40)	0.003
Age (yo)	−0.04 (−0.12, 0.04)	0.29	−0.036 (−0.11, 0.04)	0.35	−0.04 (−0.12, 0.03)	0.26
BMI	−0.05 (−0.39, 0.29)	0.77	−0.10 (−0.44, 0.25)	0.57	−0.06 (−0.40, 0.29)	0.74
CRP (mg/dL)	−1.75 (−3.26, −0.24)	0.02	−1.68 (−3.17, −0.19)	0.03	−1.77 (−3.23, −0.26)	0.02
	Dependent Variable:		Albumin			
	Model 1		Model 2		Model 3	
	β (95%CI)	*p*	β (95%CI)	*p*	β (95%CI)	*p*
Occlusal force (N)	−0.00003 (0, 0)	0.56				
Masticatory performance (mg/dL)			−0.001 (−0.002, 0)	0.02		
EAT-10					0.02 (−0.03, 0.07)	0.45
Protein intake (g)	0 (−0.005, 0.004)	0.81	−0.001 (−0.005, 0.003)	0.67	−0.001 (−0.005, 0.004)	0.81
Sex (female)	0.10 (−0.04, 0.25)	0.16	0.10 (−0.05, 0.24)	0.18	0.11 (−0.04, 0.25)	0.15
Age (yo)	0.001 (−0.004, 0.005)	0.73	0 (−0.004, 0.005)	0.89	0.001 (−0.004, 0.005)	0.76
BMI	−0.00006 (−0.02, 0.02)	0.99	0.004 (−0.02, 0.02)	0.69	−0.002 (−0.021, 0.017)	0.84
CRP (mg/dL)	−0.11 (−0.20, −0.03)	0.01	−0.12 (−0.20, −0.04)	0.006	−0.11 (−0.20, −0.03)	0.01
	Dependent Variable:		Vitamin B_12_			
	Model 1		Model 2		Model 3	
	β (95%CI)	*p*	β (95%CI)	*p*	β (95%CI)	*p*
Occlusal force (N)	−0.02 (−0.07, 0.03)	0.5				
Masticatory performance			−0.02 (−0.42, 0.39)	0.92		
EAT-10					−1.5 (−23.8, 20.7)	0.9
Vitamin B_12_ intake (μg)	12.3 (3.4, 21.2)	0.007	12.7 (3.9, 21.5)	0.005	12.6 (3.8, 21.5)	0.006
Sex (female)	86.2 (25.8, 146.5)	0.006	87.4 (27.0, 147.9)	0.005	87.5 (27.1, 147.9)	0.005
Age (yo)	3.2 (1.4, 5.1)	<0.001	3.3 (1.4, 5.2)	<0.001	3.3 (1.4, 5.2)	<0.001
BMI	−2.0 (−10.1, 6.2)	0.63	−2.1 (−10.5, 6.2)	0.61	−2.1 (−10.4, 6.1)	0.61
CRP (mg/dL)	10.9 (−24.6, 46.4)	0.54	11.9 (−23.7, 47.6)	0.51	12.1 (−23.4, 47.6)	0.5

Model 1: adjusted with Occlusal force, each nutrient (protein or Vitamin B_12_) intake, sex, age, BMI, and CRP. Model 2: adjusted with Chewing function, each nutrient (protein or Vitamin B_12_) intake, sex, age, BMI, and CRP. Model 3: adjusted with Swallowing, each nutrient (protein or Vitamin B_12_) intake, sex, age, BMI, and CRP.

**Table 4:** Multivariate linear regression analysis of chemical digestion and serum prealbumin, albumin, and vitamin B_12_ levels.

	Dependent Variable:		Prealbumin			
	Model 1		Model 2		Model 3	
	β (95%CI)	*p*	β (95%CI)	*p*	β (95%CI)	*p*
Pylori eradication	2.0 (−0.5, 4.5)	0.12				
*H. pylori* Ab			−0.12 (−0.13, 0.08)	0.65		
Oral antacids					−3.3 (−6.5, −0.1)	0.04
Protein Intake (g)	0.1 (0.03, 0.2)	0.006	0.1 (0.02, 0.2)	0.01	0.10 (0.03, 0.17)	0.007
Sex (female)	−4.0 (−6.5, −1.4)	0.003	−4.0 (−6.6, −1.4)	0.003	−4.1 (−6.6, −1.5)	0.002
Age (yo)	−0.05 (−0.12, 0.03)	0.24	−0.04 (−0.1, 0.04)	0.28	−0.01 (−0.09, 0.07)	0.8
BMI (kg/m^2^)	−0.1 (−0.40, 0.24)	0.56	−0.04 (−0.4, 0.3)	0.82	−0.01 (−0.34, 0.33)	0.97
CRP (mg/dL)	−1.6 (−3.1, −0.1)	0.034	−1.8 (−3.3, −0.3)	0.02	−1.4 (−2.9, 0.1)	0.06
	Dependent Variable:		Albumin			
	Model 1		Model 2		Model 3	
	β (95%CI)	*p*	β (95%CI)	*p*	β (95%CI)	*p*
Pylori eradication	−0.007 (−0.15, 0.14)	0.93				
*H. pylori* Ab (+)			0.002 (−0.004, 0.0089)	0.55		
Oral antacids					−0.14 (−0.32, 0.04)	0.13
Protein Intake (g)	−0.001 (−0.005, 0.003)	0.74	−0.001 (−0.005, 0.004)	0.8	−0.001 (−0.005, 0.003)	0.74
Sex (female)	0.11 (−0.04, 0.25)	0.16	0.11 (−0.04, 0.25)	0.16	0.10 (−0.04, 0.25)	0.16
Age (yo)	0.001 (−0.003, 0.005)	0.68	0.001 (−0.003, 0.005)	0.7	0.002 (−0.002, 0.007)	0.34
BMI (kg/m^2^)	−0.001 (−0.02, 0.02)	0.95	−0.001 (−0.021, 0.018)	0.88	0.001 (−0.02, 0.02)	0.93
CRP (mg/dL)	−0.11 (−0.20, −0.03)	0.01	−0.11 (−0.19, −0.02)	0.01	−0.10 (−0.18, −0.01)	0.03
	Dependent Variable:		Vitamin B_12_			
	Model 1		Model 2		Model 3	
	β (95%CI)	*p*	β (95%CI)	*p*	β (95%CI)	*p*
Pylori eradication	−48.3 (−107.8, 11.2)	0.11				
*H. pylori* Ab (+)			−1.2 (−3.6,1.2)	0.33		
Oral antacids					22.6 (−54.4, 99.6)	0.56
Vitamin B_12_ intake (μg)	12.7 (4.0, 21.3)	0.005	12.2 (3.4,21.0)	0.007	12.8 (4.0, 21.6)	0.005
Sex (female)	87.8 (28.3, 147.3)	0.004	87.7 (27.7,147.8)	0.005	88.0 (27.8, 148.3)	0.005
Age (yo)	3.4 (1.5, 5.2)	<0.001	3.3 (1.5, 5.2)	<0.001	3.1 (1.0, 5.1)	0.003
BMI (kg/m^2^)	−0.8 (−9.0, 7.4)	0.84	−1.8 (−9.9,6.3)	0.66	−2.5 (−10.7, 5.7)	0.55
CRP (mg/dL)	9.0 (−26.1, 44.2)	0.61	10.8 (−24.6,46.1)	0.55	9.7 (−26.5, 45.9)	0.6

Model 1: adjusted with history of *H. pylori* eradication, each nutrient (protein or vitamin B_12_ intake, sex, age, BMI, and CRP. Model 2: adjusted with *H. pylori* Ab + (antibody titer above 10 U/L), each nutrient (protein or vitamin B_12_) intake, sex, age, BMI, and CRP. Model 3: adjusted with oral antacids, each nutrient (protein or Vitamin B_12_) intake, sex, age, BMI, and CRP. Abbreviartion: *H. pylori*, *Helicobactor pylori*; Ab, antibody.

## Data Availability

Some or all datasets generated during and/or analyzed during the current study are not publicly available but are available from the corresponding author upon reasonable request.
